# Improved HIV-1 RNA detection using whole blood versus plasma in antiretroviral-treated individuals

**DOI:** 10.1128/jcm.01904-24

**Published:** 2025-06-04

**Authors:** Vivian I. Avelino-Silva, Mars Stone, Leilani Montalvo, Clara Di Germanio, Sonia Bakkour, Marion C. Lanteri, Eduard Grebe, Brian Custer, Xutao Deng, Renata Buccheri, Karen Harrington, Steven H. Kleinman, Sandhya Vasan, Nittaya Phanuphak, Carlo Sacdalan, Siriwat Akapirat, Mark de Souza, Esper G. Kallás, Sheila de Oliveira Garcia Mateos, Ester C. Sabino, Philip J. Norris, Michael P. Busch, for the NHLBI Recipient Epidemiology and Donor Evaluation Study-IV-Pediatric (REDS-IV-P)

**Affiliations:** 1 Vitalant Research Institute 166672 , San Francisco, California, USA; 2 Department of Epidemiology and Biostatistics, University of California San Francisco 8785, San Francisco, California, USA; 3 Faculdade de Medicina da Universidade de Sao Paulo 117265, São Paulo, State of São Paulo, Brazil; 4 Department of Laboratory Medicine, University of California San Francisco 8785, San Francisco, California, USA; 5 Creative Testing Solutions, Tempe, Arizona, USA; 6 Hologic, Inc. 315338 , San Diego, California, USA; 7 University of British Columbia, Victoria, British Columbia, Canada; 8 U.S. Military HIV Research Program, Walter Reed Army Institute of Research 8394, Silver Spring, Maryland, USA; 9 Henry M. Jackson Foundation for the Advancement of Military Medicine, Inc., Bethesda, Maryland, USA; 10 Institute of HIV Research and Innovation (IHRI) 606508, Bangkok, Thailand; 11 SEARCH Research Foundation, Bangkok, Thailand; 12 Research Affairs, Faculty of Medicine, Chulalongkorn University 65103, Bangkok, Thailand; 13 Armed Forces Research Institute of Medical Sciences (AFRIMS) 544411, Bangkok, Thailand; 14 Instituto Butantan 196591, São Paulo, São Paulo, Brazil; 15 Universidade Municipal de Sao Caetano do Sul 119501, São Caetano do Sul, São Paulo, Brazil; 16 Department of Medicine, University of California San Francisco 166668, San Francisco, California, USA; St Jude Children's Research Hospital, Memphis, Tennessee, USA

**Keywords:** HIV testing, sensitivity and specificity, nucleic acid amplification techniques, blood donation

## Abstract

Currently, nucleic acid testing (NAT) platforms detect HIV-1 in plasma. Using whole blood (WB) could improve HIV-1 detectability as cellular elements may also contain HIV-1 nucleic acids. We used well-characterized paired WB/plasma panels to evaluate HIV-1 RNA detection inhibition by WB, specificity, and enhanced HIV-1 RNA detectability by WB compared to plasma. Panels included: spiked samples; NAT−/serology−, NAT+/serology+, and NAT−/serology+ blood donor samples; samples from persons with HIV (PWH) who started antiretroviral treatment (ART) at chronic infection stages; and from PWH under ART since acute/early infection. We found one false-positive result on WB testing of 100 NAT−/serology− blood donors, and evidence of modest HIV-1 detection inhibition. Among NAT-/serology+ donors, HIV-1 RNA detectability in plasma and WB was similar (*P* = 0.64). Among 50 PWH starting ART at chronic infection stages, detectability was 24% in plasma and 92% in WB (*P* < 0.001). Among 345 PWH on ART since acute/early infection, detectability was 10% in plasma and 16% in WB (*P* = 0.013). HIV-1 RNA detectability in both plasma and WB was progressively lower for earlier Fiebig stages at ART initiation. WB increased HIV-1 detectability relative to plasma in PWH who initiated ART at all but the earliest infection stages. We failed to find enhanced HIV-1 RNA detectability by WB in NAT−/serology+ blood donors, who may include elite controllers. Enhancing HIV-1 nucleic acid detectability could improve infection ascertainment among PWH on ART with blunted serologic reactivity; investigation of breakthrough infection in PrEP users; and potentially for virus rebound monitoring in HIV-1 cure studies.

**IMPORTANCE:**

Currently, tests to detect HIV genetic materials (RNA/DNA) are done using the liquid component of a blood sample (plasma). However, HIV may be present in blood cellular components, such as white cells and platelets. Here, we investigated if using whole blood (WB; liquid + cellular components) could improve HIV RNA detectability compared to plasma. WB increased HIV RNA detectability in persons with HIV under treatment, including those with early treatment initiation, but not among blood donors with positive HIV serology and undetectable HIV RNA in the donation screening. Enhancing HIV RNA/DNA detectability would support HIV diagnosis in cases with blunted serologic response, such as persons with early antiretroviral treatment initiation or pre-exposure prophylaxis users. It would also be useful for monitoring virus rebound in HIV cure studies and in blood donation screening, where high test sensitivity is required to guarantee the safety of the blood supply.

## INTRODUCTION

HIV-1 nucleic acid testing (NAT) and viral load (VL) assays have been performed on blood specimens for decades as part of diagnostic algorithms (1), as well as for monitoring the effect of antiretroviral treatment (ART) (2, 3). Currently, available NAT assays detect HIV-1 in plasma, with analytic sensitivities varying between 10 and 100 copies/mL. However, plasma is not the only potential source for HIV-1 nucleic acid detection in blood samples. HIV-1 RNA and DNA may also be present in cellular elements, including platelets (4–6) and leukocytes (7), indicating that using whole blood (WB) specimens could improve HIV-1 detectability compared to plasma. Previous studies have shown enhanced HIV-1 detectability in WB samples from persons with HIV (PWH) under ART, with detection of HIV-1 nucleic acids in WB specimens with undetectable VL in paired plasma reported as 59.3% by Steinmetzer et al. (8) and as 96.5% by Jagodzinski et al. (9).

Several clinical settings could particularly benefit from an enhanced detectability of HIV-1 nucleic acids. These include HIV cure studies, where close monitoring of virus rebound is needed following analytic treatment interruption (10), and the diagnostic investigation of breakthrough infection in persons with prior exposure to antiretrovirals for HIV-1 pre-exposure prophylaxis (PrEP), which can lead to reduced viral replication and a blunted antibody response (11–16). Identifying laboratory evidence of HIV-1 infection may also be challenging for PWH who initiate ART at acute/early stages of infection, when antigen (Ag)-specific antibody (Ab) responses are not completely established, serologic tests may fail to become reactive or revert to nonreactive, and plasma VL may remain undetectable due to ART (16–22). In this context, using WB specimens may enhance the identification of HIV-1 nucleic acids compared to plasma, although very early initiation and long-term ART may suppress detectability even for cell-associated HIV-1 (9). Finally, blood donation screening traditionally implements the most sensitive screening assays to maximize the safety of the blood supply. Recent studies have demonstrated undisclosed use of antiretrovirals by candidate donors for HIV prevention or treatment (23–28), raising concerns about a reduced ability to identify HIV-1 positive donations, potentially impacting the risk of HIV transfusion transmission.

Here, we investigate HIV-1 detectability in samples spiked with armored HIV-1 RNA and paired WB and plasma specimens from well-characterized HIV-1 positive and negative sample panels, using commercially available, high throughput HIV-1 NAT assays adapted for testing WB samples.

## MATERIALS AND METHODS

### Study samples and panels

We used the following panels of human specimens:

1. HIV-1-negative samples spiked with serially diluted armored RNA (Hologic, San Diego, CA). The armored RNA is an *in vitro* transcribed, 1,000-base pair HIV-1 sequence encapsulated in a protein dimer coat. We tested samples unspiked; spiked with 10^1^; 10^2^; and 10^3^ copies/mL, with three replicates of each dilution. WB and plasma samples for this panel were obtained from Vitalant blood donors with negative HIV-1 NAT and nonreactive serology (NAT−/serology−). Routine Vitalant blood donation screening includes parallel testing for HIV-1/2 with a serologic test and HIV-1 NAT in minipools of 16 (detailed in the Supplemental materials).
2. Samples from 100 blood donors identified as HIV-1 NAT positive and HIV serology reactive (NAT+/serology+) at the donation screening. These samples were obtained from the Recipient Epidemiology and Donor Evaluation Study-IV-Pediatric (REDS-IV-P) Brazil program, including donations collected at five Brazilian blood centers: Fundação Pro-Sangue (São Paulo), Hemominas (Belo Horizonte), Hemope (Recife), Hemorio (Rio de Janeiro), and Hemoam (Manaus). Blood donations in these centers are routinely tested for transfusion-transmittable infections, including parallel testing for HIV-1/2 with a fourth-generation Ag/Ab combo test and HIV-1 NAT in minipools of 6.
3. Samples from 100 NAT−/serology− Vitalant blood donors (see above).
4. Samples from 367 blood donors, identified as HIV-1 NAT negative and HIV serology reactive (NAT−/serology+) during the donation screening, were obtained from the REDS-IV-P Brazil program (see above).
5. Samples from 50 ART-suppressed PWH who started ART after fully established infections (chronic), obtained from PWH followed at the University of Sao Paulo Medical School in Sao Paulo, Brazil.
6. Samples from 345 ART-suppressed PWH who started ART at acute/early stages of infection, obtained from RV254/SEARCH 010 [19] (RV254), a cohort study that offered immediate ART to persons identified with acute/early HIV-1 infection based on parallel NAT and serology screening in Bangkok, Thailand. In this panel, we analyzed overall HIV detectability in WB and plasma, as well as detectability according to the Fiebig stage (29) at ART initiation.

### HIV nucleic acid detection assays

Compared to plasma or serum, NAT of WB specimens can be inhibited by the presence of red blood cells and hemoglobin (30, 31), lactoferrin (30), and anticoagulants (32). To attenuate this inhibitory effect, we adopted a 1:4 dilution of WB samples using commercially available WB lysis reagents. Panels 1–3 were tested with a 1:4 dilution of WB using commercially available blood lysis reagents (Hologic and Grifols) using two assays: the Aptima HIV-1 Quant Dx Assay (Hologic, San Diego, CA; https://www.fda.gov/media/102425/download; Aptima) and the Procleix Ultrio Elite Assay (Grifols Diagnostic Solutions, Emeryville, CA; https://www.fda.gov/media/112861/download; Procleix). Aptima is a real-time transcription-mediated amplification (TMA) test for the detection and quantification of HIV-1 RNA groups M, N, and O, with a predicted 95% detection of 12.1 copies/mL. All HIV-1 VL quantifications for WB using Aptima are presented with a fourfold adjustment to counterbalance the dilution factor. Procleix is a qualitative TMA NAT assay that detects HIV-1 and HIV-2 RNA, hepatitis C virus RNA, and hepatitis B virus DNA, with a predicted 95% detection of 18.0 IU/mL; for this study, we applied the discriminatory component of the Procleix assay that only detects HIV 1/2 RNA. Panels 4–6 were tested with a 1:4 dilution of WB using the Aptima assay. A complete description of laboratory methods is available in the Supplemental materials.

### Statistical analysis

For each panel, we present counts and percentages of samples with positive results on plasma only, WB only, both, or neither. For panels 4–6, we describe the overall HIV-1 nucleic acid detectability on plasma or WB using percentages and 95% confidence intervals (CIs), with comparisons using chi-square tests. For tests performed on Aptima, we also describe the counts and percentages of paired samples yielding higher VL values in WB relative to plasma. For all analyses, we used Stata version 17 (StataCorp LP) with a 0.05 significance level.

purposesthe

## RESULTS

### Assay performance using HIV-1 RNA spiked samples and pedigreed negative or positive samples

**Fig 1:**
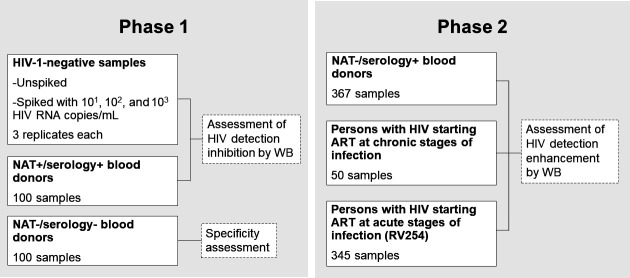
Panels and aims included in the study. Phase 1 included HIV-1-negative samples spiked with armored HIV RNA; samples from NAT+/serology+ blood donors; and samples from NAT−/serology− blood donors to assess HIV detection inhibition by whole blood and specificity. Phase 2 included samples from NAT−/serology+ blood donors and from persons with HIV under antiretroviral therapy starting treatment at chronic or acute stages of infection to assess HIV detection enhancement by WB compared to plasma.

Figure 1 shows a summary of panels and aims assessed in phases 1 and 2 of the study. In the first phase of this study, we addressed the validation of WB dilution, residual inhibition, and specificity.

First, we spiked paired plasma and WB HIV-1-negative samples from NAT−/serology− blood donors with escalating concentrations of HIV-1 armored RNA and tested samples in triplicate (Table 1). Tests performed using both assays showed that none of the unspiked replicates had detectable HIV-1 RNA in either plasma or WB. Among spiked samples, we observed progressively higher detection rates at higher concentrations of HIV-1 armored RNA and a trend toward higher detectability in plasma relative to WB, with modest evidence of HIV-1 RNA detection inhibition following WB dilution.

**TABLE 1:** HIV nucleic acid detection in paired plasma and WB samples from NAT−/serology− donors spiked with serially diluted HIV-1 armored RNA^*a*^

	Aptima		Procleix	
	Plasma VL (copies/mL)	WB VL (copies/mL)	Plasma dHIV TMA (S/CO)	WB dHIV TMA (S/CO)
1,000 copies/mL	714	303	Reactive (22.57)	Reactive (17.61)
	686	380	Reactive (22.68)	Reactive (17.76)
	822	573	Reactive (22.90)	Reactive (17.82)
100 copies/mL	53	Detected, <30	Reactive (21.92)	Nonreactive (0.30)
	94	Detected, <30	Reactive (22.16)	Nonreactive (0.16)
	60	Detected, <30	Reactive (21.92)	Reactive (13.23)
10 copies/mL	Detected, <30	0	Nonreactive (0.12)	Nonreactive (0.04)
	Detected, <30	0	Reactive (13.90)	Nonreactive (0.06)
	0	0	Reactive (20.21)	Nonreactive (0.08)
Unspiked	0	0	Nonreactive (0.04)	Nonreactive (0.04)
	0	0	Nonreactive (0.06)	Nonreactive (0.06)
	0	0	Nonreactive (0.12)	Nonreactive (0.06)

^*a*^ VL, viral load; WB, whole blood; TMA, transcription mediated amplification; S/CO, signal-to-cutoff.

**Fig 2:**
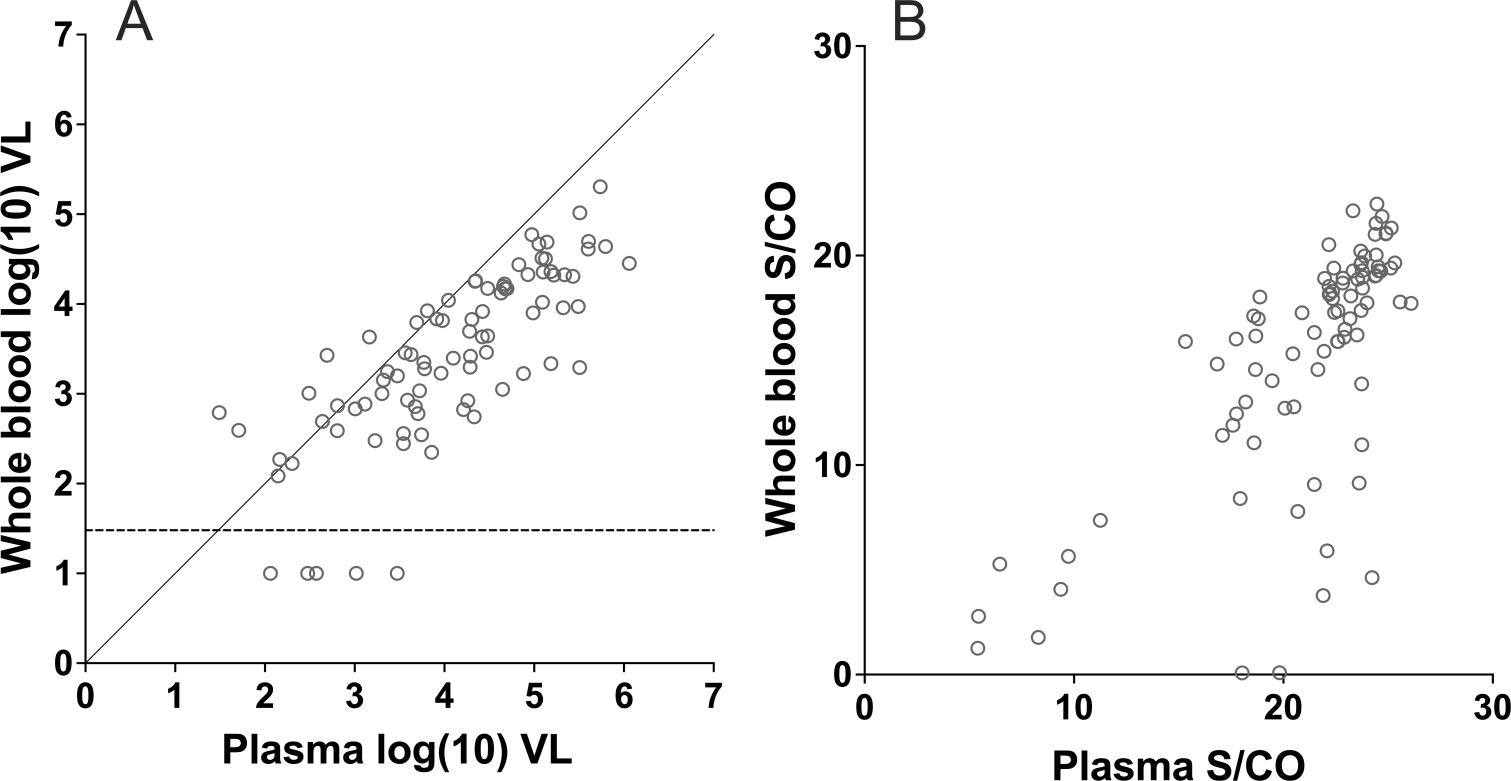
HIV-1 RNA detection in plasma and whole blood from NAT+/serology+ blood donors. (A) HIV-1 RNA viral load (VL) in plasma and whole blood using Aptima. Diagonal line indicates perfect correlation reference. (B) Signal-to-cutoff (S/CO) in plasma and whole blood using Procleix.

In the analysis of samples from NAT+/serology+ blood donations using Aptima, all 100 samples were detectable using both plasma and WB specimens. While all samples were detectable within the limit of quantification (LOQ) for plasma, seven were detectable below the LOQ in WB; VL measurement in plasma for these samples varied between 39 and 2,983 copies/mL. Although HIV-1 VL measurements in plasma and WB were highly correlated, values were higher in plasma than in WB for 86 samples (86%). Interestingly, the distribution of samples yielding higher VL measurements in WB relative to plasma was not homogenous across plasma VL measurements: of 16 samples with VL in plasma <1,000 copies/mL, 8 (50%) had higher VL measurements in WB; of 84 samples with VL in plasma >1,000 copies/mL, only 6 (7%) had higher VL measurement in WB (Fig. 2A).

Due to limited volumes, we were able to test 87 out of 100 samples from NAT+/serology+ blood donors using Procleix. HIV-1 was detected in all plasma samples and in 85 out of 87 (98%) WB samples. For the two samples that were detected in plasma and not detected in WB, plasma VL measurements by Aptima were 115 and 300 copies/mL. Signal-to-cutoff (S/CO) levels on the Procleix assay for plasma and WB are described in Fig. 2B. All except one pair had higher S/CO values in plasma relative to WB. These results show that at 1:4 used, WB did not inhibit the detection of HIV-1 RNA. At high VLs, plasma yielded higher VL readings, while at low VLs, some samples yielded higher readings in WB.

Finally, of the 100 paired samples from NAT−/serology− low-risk blood donors, testing with Aptima showed no HIV-1 detection in plasma; 8 WB samples were detectable below the LOQ, with 1 sample remaining detectable below the LOQ upon retesting. In the Procleix platform, 100/100 and 98/98 were undetectable for HIV-1 nucleic acid in plasma and WB, respectively. We obtained invalid results in the initial testing of 11 WB samples with Procleix, 2 of which remained invalid upon retesting. There was no overlap between WB samples with detectable HIV-1 NAT in Aptima and those with invalid results in Procleix.

### Assay performance in blood donors with undetectable HIV-1 in plasma and PWH on ART

**Fig 3:**
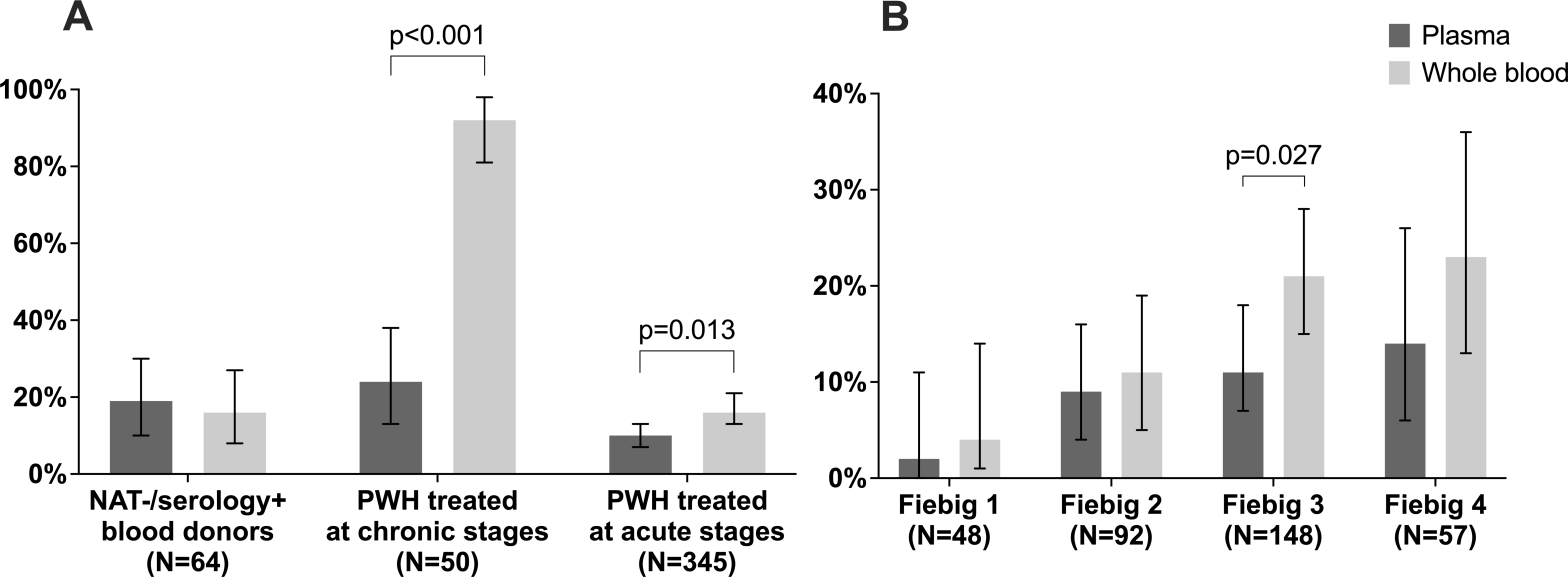
HIV RNA detectability by plasma and whole blood among persons with HIV. (A) Percentages of samples with detectable HIV RNA by group. (B) Percentages of samples with detectable HIV RNA by Fiebig stage at ART initiation among RV254 participants.

After evaluating inhibition and specificity issues for WB testing, we proceeded with the assessment of HIV-1 RNA detectability in WB relative to plasma using Aptima (Table 2 and Fig. 3A).

**TABLE 2:** Frequencies of samples with detectable HIV-1 RNA in plasma and whole blood (WB)^*a*^

		WB+ above LOQ	WB+ below LOQ	WB−	Total
NAT−/serology+ blood donors	Plasma+ above LOQ	2	4	0	6
	Plasma+ below LOQ	0	3	3	6
	Plasma−	0	1	51	52
	Total	2	8	54	64
PWH with ART onset at chronic stages of infection	Plasma+ above LOQ	0	1	0	1
	Plasma+ below LOQ	3	7	1	11
	Plasma−	12	23	3	38
	Total	15	31	4	50
PWH with ART onset at acute stages of infection	Plasma+ above LOQ	3	1	1	5
	Plasma+ below LOQ	0	6	23	29
	Plasma−	0	46	265	311
	Total	3	53	289	345

^*a*^ PWH, persons with HIV; ART, antiretroviral treatment; LOQ, limit of quantification.

Of the 367 paired plasma and WB samples from NAT−/serology+ blood donors, 309 had sufficient volume and were tested with Ortho VITROS HIV Combo (https://www.fda.gov/vaccines-blood-biologics/approved-blood-products/vitros-immunodiagnostic-products-hiv-combo-reagent-pack-vitros-immunodiagnostic-products-hiv-combo), the confirmatory Ag/Ab assay used in REDS-IV-P central lab. Of the 309 samples, 245 were nonreactive, and 64 had confirmed HIV reactive serology. Among the 245 nonreactive samples on the confirmatory assay, 1 had detectable HIV-1 RNA in WB with a VL of 131 copies/mL, with corresponding undetectable VL in plasma (data not shown). Among the 64 confirmed seropositive samples, 9 had HIV-1 RNA detectable in WB and plasma, 1 was detectable only in WB, and 3 were detectable only in plasma (all <LOQ). The overall detectability was 19% (95% CI: 10–30%) in plasma and 16% (95% CI: 8–27%) in WB (*P* = 0.639).

Among samples from 50 PWH under ART with undetectable HIV VL in routine tests who had initiated ART at chronic stages of infection, we observed that one sample had HIV-1 RNA detectable only in plasma (below the LOQ) and 35 had HIV-1 RNA detectable only in WB (23 detected below the LOQ and 12 within the LOQ, with VL varying between 123 and 1,125 copies/mL). Of the 11 samples with HIV-1 RNA detectable in both plasma and WB, 7 pairs were detected below the LOQ in both WB and plasma, 1 was detected with a VL of 134 copies/mL in plasma and below the LOQ in WB, and 3 pairs were detected below the LOQ in plasma and above the LOQ in WB, with VL varying between 199 and 257 copies/mL. The overall detectability was 24% (95% CI: 13–38%) in plasma and 92% (95% CI: 81–98%) in WB (*P* < 0.001).

Finally, among 345 paired samples from PWH from RV254, who had initiated ART at acute/early stages of infection, we observed that 24 samples had HIV-1 RNA detectable only in plasma (all below the LOQ), and 46 had HIV-1 RNA detectable only in WB (all below the LOQ). Of the 10 samples with HIV-1 RNA detectable in both plasma and WB, 6 pairs were detected below the LOQ in both WB and plasma, and 4 were detected at higher levels in plasma relative to WB. The overall detectability was 10% (95% CI: 7–13%) in plasma and 16% (95% CI: 13–21%) in WB (*P* = 0.013). Among participants starting ART at the Fiebig stages 1, 2, 3, and 4, the percentages detectable by plasma were 2%, 9%, 12%, and 14%, whereas the percentages detectable by WB were 4%, 11%, 21%, and 23%, respectively (Fig. 3B).

## DISCUSSION

Here, we investigated if WB could be used as an alternative sample type to enhance HIV-1 RNA detectability compared to plasma. In the first phase of this analysis, we found potential specificity issues and invalid results with WB, and modest evidence of HIV-1 detection inhibition by WB testing. HIV-1-negative samples from NAT−/serology− donors spiked with HIV-1 RNA had somewhat lower detectability in WB relative to plasma, and 2 out of 87 samples from NAT+/serology+ blood donors had false negative results on WB using the Procleix assay. While plasma from NAT+/serology+ blood donors yielded higher HIV-1 concentrations compared to WB for 93% of samples with VL >1,000 copies/mL, in samples with VL <1,000 copies/mL, we found that 50% of the samples yielded higher VL measurements in WB than plasma. We observed no statistically significant differences in the percentage of samples from NAT−/serology+ blood donors with detectable HIV-1 NAT in plasma or WB. However, WB testing significantly increased HIV-1 detectability relative to plasma in PWH under ART who initiated treatment at chronic stages of infection. This effect was markedly attenuated but still statistically significant in PWH treated with ART in the acute/early stages of infection from RV254.

WB testing has been adopted for diagnosis and monitoring of infectious agents, including CMV (33), Zika virus (34), West Nile virus (35), Babesia (36), and *Plasmodium* specimens (37). To attenuate the potential inhibitory effect of blood components, we adopted a 1:4 dilution of WB samples using commercially available WB lysis reagents. In tests performed using Aptima, a fourfold adjustment factor was implemented for VL readouts; however, this method may have led to missed detection in samples for which low HIV-1 VL levels became undetectable following dilution. In the spiked samples panel, the lower HIV-1 detection in WB relative to plasma was consistent with the absence of cell-associated HIV-1 RNA and with the lower plasma volume per unit (and hence lower number of HIV-1 copies) in WB, where approximately half of the sample volume is comprised of cellular components.

In the panel of NAT+/serology+ blood donor samples, the finding of higher HIV-1 VL measurements in WB than plasma almost exclusively in samples with VL measurements below 1,000 copies/mL suggests that WB may enhance HIV-1 NAT detection in samples with low HIV-1 plasma levels. This hypothesis was confirmed in the analysis of samples from PWH who initiated ART at chronic stages of infection, where WB testing led to HIV-1 detection in 35 out of 38 samples missed by plasma testing, and in samples from PWH treated at acute/early stages, where WB testing led to HIV-1 detection in 46 out of 311 samples missed by plasma testing. HIV-1 RNA detectability was lower in PWH who initiated ART at acute/early stages than in those treated at chronic stages, particularly for WB testing. Moreover, we found that participants initiating ART at earlier Fiebig stages had progressively lower HIV-1 nucleic acid detectability. This effect has been demonstrated previously (9, 38, 39) and is consistent with lower levels of cell-associated HIV-1 in PWH treated during acute/early stages of infection.

Unlike the other panels, we found no enhanced HIV-1 RNA detectability by WB testing compared to plasma in samples from NAT−/serology+ blood donors. It is likely that this group included both virologically suppressed PWH under ART with undisclosed HIV-1 infection status at donation and ART-naïve PWH with elite control of HIV-1 replication. For the latter group, previous reports suggest the lower levels of cell-associated HIV-1 (40), which could partially explain our findings.

Dried blood spot samples, which contain WB components, have been used for both qualitative and quantitative testing of HIV nucleic acids, with particular relevance in resource-limited settings. Interestingly, studies have shown that DBS may over-quantify HIV-1 VL when plasma VL is <1,000 copies/mL (41), consistent with our findings and potentially reflecting the detection of cell-associated HIV RNA and DNA. The potential increase in HIV nucleic acid detection in dried blood spot samples relative to plasma should be further investigated.

Our study had limitations. Samples from RV254 had been collected and stored several years ago, with storage time surpassing the manufacturers’ sample stability claims. We were unable to test for the presence of antiretrovirals in samples from NAT−/serology+ blood donors, which could help discriminate elite controllers from ART-suppressed PWH. The enhanced detectability by WB testing compared to plasma observed in panels 4 and 5 may have been partially influenced by false positive results with Aptima, which occurred in approximately 1% (95% CI: 0–5%) of tested samples according to our specificity analysis. It is unknown if these results can be generalized to other collection systems and/or other manufacturers’ HIV RNA or DNA assays. We included samples from PWH in Brazil, where subtype B is the most prevalent (42), as well as samples from Thailand, where CRF01_AE is the predominant subtype (43). While HIV subtypes have not been characterized in samples from Brazilian participants, samples from RV254 participants have been previously evaluated by gene sequencing and/or multiregion hybridization assay, confirming the recombinant form CRF01_AE in 76% of the participants. It is unknown if our results can be generalized to other HIV subtypes. Finally, we used NAT assays that do not detect HIV-1 DNA, a potentially relevant component of cell-associated nucleic acids. Data from assays capable of detecting both HIV-1 RNA and DNA are critical to understand the applicability and broader adoption of WB testing.

Despite these limitations, our study demonstrates a potential for WB to be used as an alternative sample type with enhanced HIV-1 RNA detectability compared to plasma. Additional validation steps would be required before WB testing could be considered an alternative to the Aptima and Procleix assays, including further assessment of specificity, evaluation of enhanced HIV detection relative to plasma in a larger number of samples from PWH under suppressive antiretroviral therapy, and development of strategies to avoid invalid results in WB testing. An enhanced detectability of HIV-1 nucleic acids would be advantageous for several clinical and research circumstances, including HIV-1 infection status ascertainment among PWH under ART with blunted serologic reactivity (e.g., children exposed to perinatal transmission prophylaxis and persons exposed to PrEP with breakthrough HIV-1 infection, and potentially for monitoring of virus rebound following analytic treatment interruption in HIV cure studies, although detection of HIV-1 RNA in WB may be due to the presence of replication-defective proviruses (44, 45). Furthermore, enhanced HIV-1 detectability could be valuable in settings where high sensitivity for the detection of HIV-1 nucleic acids is desirable, including blood donation screening.

## Data Availability

The data sets used and/or analyzed during the study are available from the authors on reasonable request.

## SUPPLEMENTAL MATERIAL

Supplemental materials jcm.01904-24-s0001.docx Additional details.

ASM does not own the copyrights to Supplemental Material that may be linked to, or accessed through, an article. The authors have granted ASM a non-exclusive, world-wide license to publish the Supplemental Material files. Please contact the corresponding author directly for reuse.
