# Supplementary material for: Improved HIV-1 RNA detection using whole blood versus plasma in antiretroviral-treated individuals
Source: J Clin Microbiol. 2025 Jun 4;63(7):e01904-24. doi: 10.1128/jcm.01904-24 (PMC12239725; doi:10.1128/jcm.01904-24)
Supplement: Supplemental materials — Additional details. [file jcm.01904-24-s0001.docx]

**Improved HIV-1 RNA Detection Using Whole Blood Versus Plasma in Antiretroviral-Treated Individuals**

**Vitalant routine blood donation screening for HIV-1/2**

Routine Vitalant blood donation screening is performed at Creative Testing Solutions (CTS) and Vitalant includes parallel molecular and serology testing for HIV-1/2 using the Procleix® Ultrio Elite assay which is an HIV/HBV/HCV multiplexed NAT assay (Grifols Diagnostic Solutions Inc., Emeryville, CA) on minipools of 16 and with the GS HIV-1/HIV-2 plus O enzyme immunoassay (Bio-Rad Laboratories, Redmond, WA) on the Ortho Verseia ® Integrated Processor (VIP) or, more recently, the Alinity s HIV Ag/Ab Combo chemiluminescent microparticle immunoassay which is a 4th generation antigen (Ag)/antibody (Ab) combo assay test (Abbott Laboratories, Abbott Park, IL).

**Laboratory methods for whole blood testing**

**Sample preparation**

For whole blood testing a specimen aliquot tube was prepared containing 1.5 mL of Blood Transport Medium (Hologic) or Parasite Transport Medium (Grifols). Frozen whole blood was thawed and gently mixed by pipetting up and down at least three times prior to transfer of 500 μL to the specimen aliquot tube. The tube was vortexed at least 5 seconds before testing. For plasma testing frozen plasma was thawed and mixed by pipetting up and down at least three times before transferring at least 700 μL to a specimen aliquot tube. The sample was vortexed and centrifuged at 2500 x g for 10 minutes just before testing.

Armored RNA (Hologic) was spiked into 2 mL of thawed blood donor whole blood and plasma at concentrations of 1000, 100 and 10 copies/mL. Unspiked samples were also prepared for each sample type. To qualify the diluents as negative for HIV, 10 replicates of the diluents were tested using the Aptima HIV-1 Quant Assay before use. To stabilize RNA, 3 mL of Blood Transport Medium (Hologic) or Parasite Transport Medium (Grifols) was added to a tube before adding 1 mL of whole blood. The spiked whole blood lysates were vortexed for at least five seconds. Both whole blood lysates and plasma were stored at -80°C prior to batch testing. Plasma samples were vortexed and spun at 2500 x g for 10 minutes before testing. The lysed whole blood was vortexed but not centrifuged before testing.

**HIV RNA testing**

The Aptima HIV-1 Quant Assay is a transcription mediated amplification (TMA) for the quantification of HIV-1 RNA groups M, N, and O. (Hologic). The assay uses multiple long primers that targets independently two regions, pol and LTR, of the HIV genome. TMA utilizes two enzymes, MMLV reverse transcriptase and T7 RNA polymerase. The reverse transcriptase is used to generate a DNA copy (containing a promoter sequence for T7 RNA polymerase) of the target sequence. T7 RNA polymerase produces multiple copies of RNA amplicon from the DNA copy template. The Procleix Ultrio Elite Assay is a qualitative TMA assay used for detection of HIV RNA, hepatitis C RNA, and hepatitis B DNA (Grifols). Both assays are performed on the fully automated Panther® platform using 0.5 mL of the sample. Both assays include an internal control for monitoring assay performance and utilize target capture to increase assay sensitivity.
